# Market Segmentation and Green Development Performance: Evidence from Chinese Cities

**DOI:** 10.3390/ijerph20054411

**Published:** 2023-03-01

**Authors:** Xuebing Dong, Benbo Liang, Haichao Yu, Hui Zhu

**Affiliations:** 1 China Academy of West Region Development, Zhejiang University, Hangzhou 310058, China; 2 Institute of New Structural Economics, Peking University, Beijing 100091, China; 3 School of Economics, Zhejiang Gongshang University, Hangzhou 310018, China

**Keywords:** market segmentation, green development performance, factor price distortion, industrial structure upgrading, dynamic spatial panel model, heterogeneity analysis

## Abstract

This study is based on 2006–2019 panel data from 282 Chinese cities. Market segmentation and green development performance are empirically investigated to examine their non-linear relationship using static panel, dynamic panel, and dynamic spatial panel models. The results reveal the following: (1) Green development performance is found to have a high degree of temporal and spatial path dependence, exhibiting spatial linkage between cities. (2) Market segmentation stemming from local government protection has a clear inverted U-shaped structure in relationship with the green development performance. (3) Our analysis suggests that the upgrading of industrial structures significantly enhances green development, while factor price distortion inhibits it. The relationship between market segmentation and industrial structure upgrading is also an inverted U-shape. (4) The analysis further reveals that market segmentation has an inverted U-shaped correlation with the green development performance in western, central, and eastern cities. However, the different rates of development of industrial structures within the three regions result in varying degrees of market segmentation according to inflection point values. Moreover, aligned with the theoretical hypothesis of “resource curse,” in resource-based cities (exclusively), market segmentation still affects the green development performance with a significant inverted U-shaped structure.

## 1. Introduction

An enterprise’s emissions of carbon and other waste products depend on its generation and treatment of pollutants. On the one hand, refining energy efficiency and green technology lowers this emission rate by optimizing the production process. The study found that the misuse of enterprise resources due to local market preservation reduces productivity and increases the emissions of industrial SO_2_, wastewater, fumes, and dust [1]. On the other hand, trade openness and an appropriate business environment, allowing for enterprises to freely enter or exit the market, can optimize resource allocation, improve productivity, and reduce pollutants [2]. With the rapid development of China, urbanization and industrialization have been swiftly promoted, and the problem of pollutant emissions has become increasingly serious. According to the latest data from the World Bank, China’s total carbon emissions were more than 1/3 of the global total at 10.7 billion tons in 2019. China’s export-oriented economic model is overly dependent on resource consumption, resulting in serious environmental pollution. Therefore, in order to deeply participate in global climate governance and continuously improve its ecological impact, China must vigorously advocate for the use of clean energy and low-carbon emission measures for the purpose of promoting sustainable, low-carbon, green economic development.

Market segmentation has both natural and man-made factors. The former includes climate and geographic features, etc., and the latter includes administrative and corporate market power and other monopolistic factors. The study focuses on the effect of local governmental restrictions on the influx of foreign resources and the outflow of local resources. In the interest of protecting a country’s local market, the government may intervene by economic or administrative means, thus causing commodity or factor market fragmentation [3]. Since China’s economic reform and development, the implementation of fiscal decentralization has furthered the administrative powers of local governments and enhanced their incentive to promote local economic growth. The predominant view is that, while market segmentation can temporally promote economic growth by increasing the degree of industrial isomorphism, in the long run, it would undermine overall economic growth and may also cause environmental pollution. Therefore, reducing inter-regional industrial isomorphism is an effective way to eliminate segmentation and achieve market integration [4]. Furthermore, the current and future growth of local economies is subject to an inverted U-shaped effect from segmented markets [5]. Green development performance is assessed through the evaluation of urban carbon emission and pollutants, as well as of economic growth. Thus, the main goals of this study are to answer the following questions: (1) What are the spatial distribution characteristics of green development under the influence of market segmentation in the context of China’s target of carbon peak and carbon neutrality? (2) Can breaking down local market segmentation, accelerating market integration, and building a large national unified market promote low-carbon economic development?

In terms of low-carbon development and energy saving, an important means is the optimization and upgrading of industrial structure [6]. Noticeably, in the upgrading of industrial structure, within the economic weight of agriculture, industry accounts for a lower proportion of energy saving, and the proportion of tertiary industries continues to rise, which can effectively reduce the energy consumption and industrial development brought about by pollution emissions, all while driving high economic growth. In addition, industrial structure upgrading allows for the optimal allocation of resource factors and the further improvement of development quality. Hence, in the context of there being an urgent need in China for economic development mode transformation and further supply-side structural reform, optimizing industrial structure is still considered by Wang et al. (2019) [7] as one of the key directions for restructuring, emission reduction, and energy conservation in China. In addition, from the point of view of the three driving forces of economic growth, “investment, exports, and consumption,” local governments’ intervention and control of factor markets such as land, capital, and labor are motivated by their pursuit of GDP growth and the performance assessment of government officials. Market integration is intended to eliminate market segmentation, break down inter-regional trade barriers, and effectively reduce the cost of intermediate goods trade for enterprises. The scale effect generated by the expansion of the market size further reduces the transaction costs faced by enterprises in making decisions, while promoting a greater, free, and rapid flow of the factors of production to the sectors with the highest marginal returns and the upgrading of regional industrial structures, thereby increasing the total factor productivity (TFP) and economic growth [8,9]. Regional innovation efficiency can be significantly improved through regional cooperation [10]. Therefore, most argue that green economic growth can be significantly promoted by eliminating market segmentation, and that its mechanism of action not only accelerates the cross-regional free flow of factors to optimize resource allocation, but also reduces regional industrial isomorphism, promoting industrial structure upgrading and thus increasing the TFP, which, in turn, promotes green economic growth [11,12]. Hence, can market segmentation affect the green development performance through industrial structure optimization and resource allocation with these factors? Is there a non-linear relationship?

In order to answer the above questions, this study uses Chinese city data to construct a market segmentation index, and incorporates both urban carbon emission levels and industrial pollution emissions into the green development performance evaluation frame-work. The impact of market segmentation on the green development performance is empirically tested using a spatial econometric model, whereby its spatial spillover effects and dynamic effects are explored. The influence mechanisms of industrial structure and factor distortion are analyzed to provide empirical evidence for the promotion of market integration and elimination of market segmentation to improve the regional green development performance. We make the following contributions to the existing literature: (1) Previous studies have focused on the negative effects of market segmentation on carbon emission levels and environmental pollution. However, this does not take into account that market segmentation is a catalyst for short-term regional economic growth. This study not only discusses the non-linear relationship between market segmentation and green development performance, but also explores the influence of industrial structures and resource allocation. This provides new empirical evidence for the effect of market segmentation on green development. (2) Most existing studies construct a Chinese inter-provincial market segmentation index to study the impact of market segmentation on environmental pollution. China’s provinces are vast and there is a large degree of segmentation among the provincial cities. However, this cannot explain the variability in productivity and environmental performance that occurs among different cities within the same province. China has a long history of province-based governance, and the market segmentation between provinces can be influenced by local cultural differences. Therefore, constructing a market segmentation index based on the city level can effectively reduce the effect of a high overlap between dialect distribution and the administrative divisions. This helps to analyze the environmental effects of market segmentation from a microscopic perspective. (3) Crucially, market integration breaks down local protection, eliminates market segmentation, and enables resources to flow on their own to achieve the optimal allocation, which enhances the breadth and depth of the economic activity linkages between regions [13,14]. Ignoring the temporal –spatial effects can lead to estimation bias when considering the path dependence of the green development performance. Therefore, a spatial econometric model is used herein to facilitate the analysis of the impact of market segmentation on the green development performance, as well as its temporal–spatial and dynamic spatial spillover effects, for the purpose of obtaining robust empirical analysis results.

The remaining parts of this paper are organized as follows: Section 2 presents a literature review and the research hypotheses. Section 3 describes the methodology and data. The empirical results and discussion are provided in Section 4, while Section 5 concludes the paper and relates some policy implications.

## 2. Literature Review and Research Hypotheses

Previous studies on the impact of market segmentation on green low-carbon development focus on three main areas: (1) Market segmentation affects environmental performance through economic efficiency. In China’s context, which is featured with high-level market segmentation, highly profitable industries have been developed by local governments by setting up interregional trade barriers and other means that distort factor prices and lead to domestic regional development, away from comparative advantage [3]. (2) The impact of regional trade barriers due to market segmentation on the environmental performance is studied from a microscopic perspective. Removing these trade barriers can significantly contribute to overall industry productivity growth and welfare gains. The reason for this is that intra-country trade barriers drive high-productivity firms into export markets and force the low-productivity firms out of the market [15]. Trade selection effects can induce the reallocation of production factors, preventing the pollution of clean enterprises, thus reducing the overall environmental pollution levels [16]. The levels of market segmentation affect China’s performance in energy use and carbon emissions. By eliminating market segmentation, promoting regional integration in China can significantly contribute to a better performance in energy efficiency and carbon emissions, which is beneficial to environmental improvement [17]. (3) Some studies have examined how local government protection affects the environmental performance. Under the decentralized system, local governments have a higher taxation power and administrative power, and are highly likely to lower the environmental standards to attract high-pollution and energy-consuming enterprises to boost investment, raise taxes, and increase employment opportunities. However, the decentralized system leads to vicious competition among local governments, intensifies local protection, focuses on short-term growth goals at the expense of long-term development, lowers environmental regulatory standards, and hinders the improvement of environmental performance [18,19,20].

Most of the existing studies conclude that there is a prohibiting effect of market segmentation on the improvement of environmental pollution. However, the evaluation of the green development performance herein is performed via an input–output perspective, considering both GDP as the desired output, and environmental pollutant emissions and carbon emissions as the non-desired output, which includes both economic growth and green low-carbon aspects. (1) The contribution of market segmentation to local economic growth increases with each degree before reaching a specific threshold, but when there is a high-than-threshold degree of market segmentation, the economic growth will be negatively affected [5]. (2) Market segmentation can promote regional economic growth at a certain level in the short term. According to the EKC (Environmental Kuznets Curve) model [21], when the level of economic development reaches an inflection point, with further economic growth, the level of environmental pollution gradually slows down, environmental quality gradually improves, and the green development performance increases. As the degree of market segmentation continues to expand, the regional resource allocation and factor price distortions further intensify, at which point the market segmentation can significantly inhibit the regional economic growth. According to the EKC model, the level of economic development keeps moving closer to the left side of the inflection point, and the green development performance starts to decrease. (3) Studies have found that the distortion of resource allocation also increases when there is an increasingly higher level of market segmentation, which, in turn, exacerbates the deterioration of the environmental quality and inhibits the improvement of the total factor productivity and energy efficiency [22]. Therefore, when considering the simultaneous inclusion of economic growth, environmental pollution, and carbon emissions in the performance evaluation system, the first hypothesis is presented.

**Hypothesis 1.**  *A certain degree of market segmentation promotes local economic growth, but when the threshold is exceeded, it inhibits economic growth and aggravates environmental degradation; thus, there is an inverted U-shaped structure regarding the impact of market segmentation on green development performance.*

Industrial structure upgrading refers to a process in which traditional high-energy-consuming and highly polluting secondary industries are gradually replaced with low-energy-consuming tertiary industries, such as the technology and service industries, which enables economic development while reducing environmental pollution and carbon emissions, thus promoting the improvement of regional green development performance [11,23]. First, the upgrading of this industrial structure promotes resource allocation optimization, thus making full use of these resources to reduce environmental pollution [24]. Moreover, it indirectly improves China’s urban green emission reduction by promoting technological change in order to improve resource efficiency [25]. Second, by strengthening the public and non-public sector cooperation among local governments, the intensity of technology spillover increases, thus boosting industrial structure optimization and upgrading [26]. Third, market segmentation hinders the allocation of resources and product flows between the regions of China, causing intra-regional competition and the duplication of construction, inhibiting the improvement of market specialization and the expansion of economies of scale, making production patterns deviate from comparative advantage [27]. It also hinders foreign firms from investing in local firms while hindering the productivity-boosting effect of technology spillovers on local firms. Furthermore, when market segmentation is at a higher degree, its hindering effect is greater [28]. Finally, the lower intensity of market segmentation promotes the productivity of local firms by providing them with protection and incentives, among other measures. However, with the increasing intensity of market segmentation, local enterprises, especially those with imperfect internal governance mechanisms, can gain monopoly profits without transformation and upgrading due to local protection, meaning that the possibility of enterprises resting on the status quo and lacking enterprising spirit gradually increases, which, in turn, hinders the occurrence of productivity-enhancing behaviors such as R&D, technology introduction, and achieving economies of scale. Market segmentation, when at a higher degree, tends to have a more significant negative impact on the transformation of production that offsets its positive impact [29]. Therefore, hypothesis 2 is proposed.

**Hypothesis 2.**  *Market segmentation affects the regional green development performance by influencing the level of advanced regional industrial structure. It has both positive and negative effects on enterprise productivity improvement, transformation, and upgrading. When market segmentation is at a greater degree, its negative effect is more likely to offset its positive effect, so the impact on regional industrial structure upgrading also shows an inverted U-shaped structure.*

According to neoclassical economics, under the condition of a perfectly competitive market, factor prices are equal to their marginal output values, and the market can automatically achieve equilibrium at this price level, while there can be no price distortion in both product and factor markets. However, the deviation or divergence between the market price of the factors and their opportunity cost, due to the non-optimal allocation of factor resources in the national economy caused by market imperfection, is the factor price distortion [30]. (1) The distortion of the price of factors makes high-energy-consuming and high-polluting enterprises obtain excessive profits through lower labor and capital costs, and serves to further expand their production scale and increase their pollution emissions [31]. (2) The low-cost advantage brought about by the distortion of the elemental market to the enterprises is transformed into their export advantage, which promotes the export scale of non-technology-intensive, low-end products and intensifies environmental pollution [32]. (3) Factor market distortions inhibit energy efficiency improvements and corporate R&D technological advances, which, in turn, exacerbate the regional environmental pollution and carbon emission levels [33,34]. (4) The market segmentation stemming from local protection has not only severely weakened the market’s ability to allocate resources by restricting the flow of factors and commodities, but has also led to the continuous distortion of factor market prices [3,35]. Therefore, hypothesis 3 to be tested herein is proposed.

**Hypothesis 3.**  *Market segmentation is detrimental to green development performance by continuously increasing factor price distortion, which would then affect the efficiency of factor resource allocation.*

## 3. Methodology and Data Description

### 3.1. Benchmark Model

The following benchmark model is constructed to identify how the green development performance is affected by market segmentation:

(1) $$Cete_{it}=\eta_{0}+\alpha_{1}Mseg_{it}+\alpha_{2}Mseg_{it}{}^{2}+\beta X_{it}+\varphi_{i}+\gamma_{t}+\mu_{it}$$

where *i*, *t* stand for city and year, respectively, $Cete_{i,t}$ is the green development performance, $Mseg_{it}$ is the degree of urban market segmentation, $Mseg_{it}{}^{2}$ represents the secondary term for the degree of urban market segmentation, $X_{it}$ is the related control variables, $\alpha_{1}$, $\alpha_{2}$ and β are the coefficients of the market segmentation and control variables affecting the green development performance, respectively, $\eta_{0}$ is the intercept term, $\varphi_{i}$ is an individual urban-fixed effect, $\gamma_{t}$ is a time-fixed effect, and $\mu_{it}$ is the random error term.

To test hypotheses 2 and 3 and verify the impact mechanism, the mediating effects model is constructed upon the baseline model (1) [36].

(2) $$M_{it}=\eta_{1}+\alpha_{3}Mseg_{it}+\alpha_{4}Mseg_{it}{}^{2}+ⱱZ_{it}+\varphi_{i}+\gamma_{t}+\mu_{it}$$

(3) $${Cete}_{it}=\eta_{2}+\alpha_{5}Mseg_{it}+\alpha_{6}Mseg_{it}{}^{2}+\omega M_{it}+{ⱱ}_{2}Z_{it}+\varphi_{i}+\gamma_{t}+\mu_{it}$$

$M_{it}$ are mediating variables (the level of advanced industrial structure and the degree of the factor price distortion), $Z_{it}$ is a set of control variables in addition to the mediating variables, $\varphi_{i}$ and $\gamma_{t}$ are urban individual fixed effects and time-fixed effects, respectively, and $\mu_{it}$ is the random error term. In particular, $\alpha_{1}$ and $\alpha_{2}$ are both significant, so as to support the existence of the mediating effect. Additionally, when $\alpha_{3}$, $\alpha_{4}$, and ω are all significant, if $\alpha_{5}$ and $\alpha_{6}$ are both significant but the coefficients are reduced, it is then considered to be a partial mediating effect. If neither $\alpha_{5}$ nor $\alpha_{6}$ are significant, then it is a fully mediated effect.

### 3.2. Dynamic Panel and Dynamic Spatial Panel Models

Changes in the economic variables inherently have some path dependence. The results of the previous period tend to have some influence on the results of the later period, and the carbon emissions and environmental pollution in Chinese cities may have a spatial and temporal lag. Drawing on [37], dynamic panel and dynamic spatial panel models are constructed by introducing spatially lagged and time-lagged terms of the explanatory variables to portray the spatial and temporal effects of the green development performance.

(4) $$Cete_{i,t}=\eta_{0}+\tau Cete_{i,t-1}+\theta_{1}Mseg_{i,t}+\theta_{2}Mseg_{i,t}{}^{2}+\beta X_{i,t}+\varphi_{i}+\gamma_{t}+\mu_{i,t}$$

(5) $$Cete_{i,t}=\eta_{0}+\tau Cete_{i,t-1}+\Psi WCete_{i,t-1}+\rho WCete_{i,t}+\theta_{3}Mseg_{i,t}+\theta_{4}Mseg_{i,t}{}^{2}+\beta X_{i,t}+\varphi_{i}+\gamma_{t}+\mu_{i,t}$$

where *i, t* are the city and year, respectively, W is the spatial weight matrix, $Cete_{i,t-1}$ is a time lag term for the green development performance, $WCete_{i,t}$ and $WCete_{i,t-1}$ are the spatial lag term and the temporal–spatial lag term of the green development performance, respectively, $\varphi_{i}$, $\gamma_{t}$, $\mu_{i,t}$ are the individual fixed effects, time-fixed effects, and random error terms, respectively, τ and Ψ are the coefficients of the time lag effect, as well as the space–time lag effect, respectively, and ρ is the spatial correlation coefficient.

For the explanatory variables, the spatial econometric model point estimates further calculations of their global direct and indirect effects, which can be used to characterize their effect on the explained variables [38]. Since the dynamic spatial panel model also includes time lagged terms and the time lagged term of each explanatory variable, the calculated global direct effects, as well as the indirect effects, are also differentiated into long-run and short-run effects, which better portray the effect that the explained variables receive from the explanatory variables at the temporal and spatial levels. In addition to reporting the point estimates of the model, this study also additionally calculates the direct and indirect effects of all the explanatory variables on green low-carbon development.

### 3.3. Spatial Weight Matrix

In this study, the dynamic spatial panel model is estimated mainly using the spatial weight matrix as follows. 

W1: The inverse distance spatial weight matrix of the shortest cross-city driving distance in the center of mass crawled by AMAP is used. The further the driving distance between the two cities, the smaller the spatial weight coefficient, representing a lower connection between the two places. Compared with the previous straight-line distance between the centers of mass calculated by the projected geographic coordinates of the cities, the driving distance is more in line with the reality of the factor and commodity flows between the urban areas. However, due to the strong exogeneity of the distance matrix of urban geographic locations, it is a good circumvention for the endogeneity of the spatial weight matrix.

W2: The geographical binary adjacency matrix.

W3: The spatial weight matrix of the sum of the geographic economies (Case et al. 1993) [39], where $W_{3}=\Phi W_{1}+\left(1-\Phi \right)W_{\mathrm{ij}}$, $W_{ij}=\left[1/\left|emp_{i}-emp_{j}+1\right|\right]*e^{-\beta d_{ij}}$, $W_{\mathrm{ij}}$ is the inverse economic distance spatial weight matrix, and β is 100, $d_{ij}$ is the distance between region *i* and region *j* (Jeanty 2014) [40]. In this study, the Φ reference (Jia et al. 2021) study is taken as 0.5 [41].

W4: The spatial weight matrix of the inverse distance between the two geocentric spherical surfaces projected in the geographic coordinates.

### 3.4. Data Description

#### 3.4.1. Explained Variables (Cete): Green Development Performance

In this study, we select the urban capital stock as the capital input [41]. The quantity of the individuals employed in the urban units in each city at the year-end is selected as the labor input [25]. The capital stock is estimated using the “perpetual inventory method” [42]. The calculation method of IPCC (2006) is used to calculate the carbon emissions of the urban non-desired outputs. The annual electricity usage, total gas supply, and LPG supply are converted by corresponding the standard coal conversion factors to a million tons of standard coal, and they are then added to get the energy input of each city [43]. The desired output is the GDP published in the China Urban Statistical Yearbook, converted to constant 2003 prices. Using Chinese urban industrial wastewater, industrial SO_2_, and industrial soot emissions as the non-desired output of environmental pollution, Input and output variables in Table 1, the green development performance is measured by employing the highly efficient SBM-DEA model [44]. 

#### 3.4.2. Core Explanatory Variables (Mseg): Market Segmentation

When it comes to China, the level of market segmentation has been measured using five main methods: the economic cycle method, the output structure method, the trade flow analysis method, the technical efficiency method, and the price method [5]. However, due to the city data available, this study measures the degree of city market segmentation by the proportion of domestic trade to the GDP of each city. Domestic trade is also replaced by the total retail sales of consumer goods, as this avoids price factors such as inflation, with the city-level data being relatively complete and having a good fitting effect [45,46].

#### 3.4.3. Control Variables

From the perspectives of socio-economic development, environmental protection, and pollution emission, the following control variables are selected for this study to minimize the bias of the estimation results caused by any omitted variables.

(1) Wage level (lnaw). This is expressed by using the average wage of urban workers to take the logarithm [47].
(2) Urbanization level (ps). This is expressed by using the urban population as a share of the total population, as an indicator of urbanization [48].
(3) Fiscal decentralization (fd). This is expressed by using the ratio of the per capita fiscal expenditure in the city to the sum of central, provincial, and urban per capita fiscal expenditure [49].
(4) Environmental Regulation (er). This is expressed by extracting the proportion of the frequency of words involving environmental protection in the work reports released by each urban government (environmental word frequency specifically includes air, PM10, PM2.5, sulfur dioxide, carbon dioxide, low carbon, emission reduction, emissions, pollution, environmental protection, ecology, green, and energy consumption.) in China from 2006–2019, to the total number of full-text words, to indicate the strength of the environmental regulation of local governments [11].
(5) Openness to the outside world (oul). This control variable is expressed by using the proportion of the actual foreign investment that is utilized by cities to the regional GDP [46].
(6) Technology Innovation Level (lnsqt). This is expressed by using the logarithm of the number of patent applications [11].
(7) Government regulation ability (gov). This is expressed by using a government spending to GDP ratio to measure the local government’s ability to intervene in the economy [7].
(8) Population size (lnpop). This is expressed by using China’s urban year-end household population taken as log [50].
(9) Infrastructure level (infs). This is expressed by adopting the measurement of the urban road area per capita, and the average road area per person is calculated according to the year-end household registration population in urban areas [51].
(10) Economic Development Level (lnprgdp). This is expressed by using a logarithm of real GDP per capita [51].
(11) Human capital level (edu). This is expressed using the number of students enrolled in higher education per 10,000 people [52].

#### 3.4.4. Mechanism Variables

(1) The degree of the distortion of elemental prices (dist). This study draws on the approach of (Hsieh et al. 2009) [53] to calculate factor price distortions, using the production function approach with input selection capital and labor. The production function considers the general choices beyond the log production function and estimates the output elasticity coefficients of capital and labor, using a double fixed effects panel regression (please contact the authors for the calculation process of the price distortion index.). The average of the calculated labor and the capital price distortions is taken as the overall factor distortion. Considering that there are positive distortions (the marginal factor output is less than the real cost) and negative distortions (the marginal factor output is greater than the real cost) in the factor market, we consider that the factor allocation reaches the optimal level when the real cost of the factor is equal to the marginal output. Therefore, the factor distortion index is subtracted by 1 and the absolute value is taken to measure the deviation of the factor market from the optimal allocation. A larger value suggests that the factor is further from the optimal allocation and the degree of factor distortion is higher.

(2) Industrial structure upgrading (ts). In this study, the advanced level of industrial structure is represented by the proportion of the added value of the tertiary industries to that of the secondary industries, and a larger value suggests that regional industrial structure has a higher level of upgrading [54].

### 3.5. Data Sources and Characteristic Facts Analysis

#### 3.5.1. Data Source

The statistical descriptions of the variables above are shown in Table 2. In this study, the statistical data of 282 cities in China from 2006–2019 are selected, and the data are mainly obtained from the China City Statistical Yearbook, China Energy Statistical Yearbook, and the statistical bulletin of each city.

#### 3.5.2. Spatial Correlation Test

The spatial relevance of the green development performance needs to be examined before conducting a spatial econometric analysis. In this study, the local and global Moran’s I scatter plots are used to examine the spatial correlation of the variables. The spatial weight matrix is W1.

**Figure 1:**
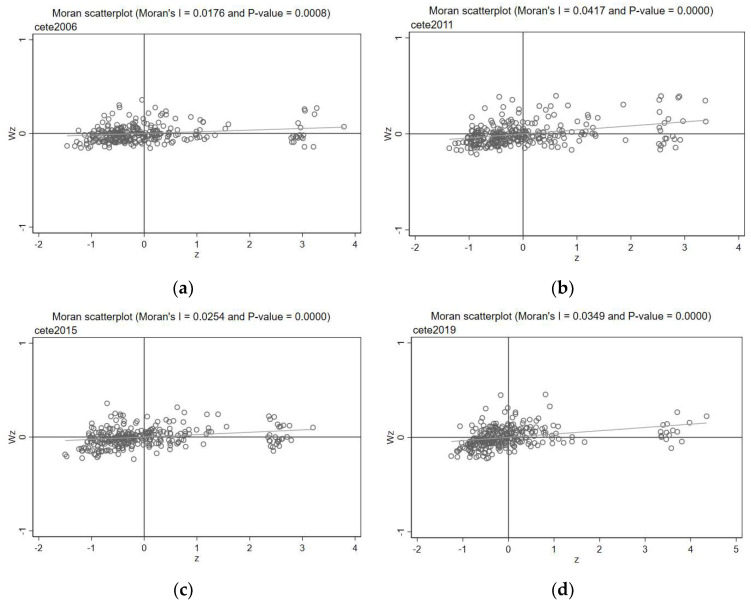
Moran Index and scatter plot of green development performance of Chinese cities in 2006 (**a**), 2011 (**b**), 2015 (**c**), and 2019 (**d**).

**Figure 2:**
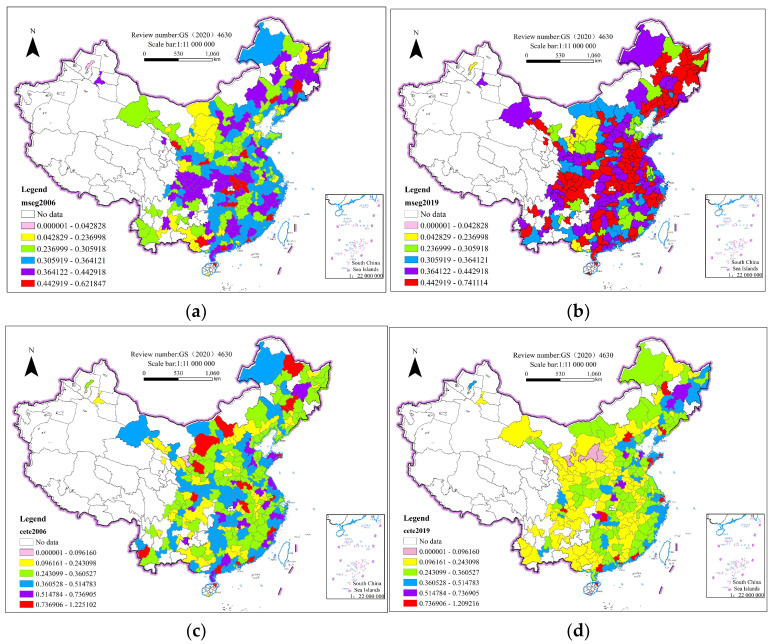
Spatial and temporal quantile of green development performance of 282 cities in China. (**a**): Mseg2006; (**b**): Mseg2019; (**c**): Cete2006; (**d**): Cete2019.

Table 3 is a global Moran index of the green development performance for each year from 2006–2019. At the 1% significance level, the Moran index for each year is significantly positive. This indicates that hypothesis 1, regarding the positive spatial spillover effect of the green development performance, holds. Our model is selected according to the Akaike Information Criterion (AIC), Bayesian Information Criterion (BIC), and the magnitude of the log-likelihood value. The smaller values of AIC and BIC, and the larger values of log-likelihood, indicate better models. The results of a regression analysis on each model in Table 3 showed that the SAR model was the best. Due to the Hausman test results in Table 3, the original hypothesis of using the random effect model is rejected, and the fixed effect model is selected. To avoid the estimation bias caused by the regional and time differences, we estimate the temporal–spatial parameters using the two-way fixed effects of the SAR model in our spatial measurement calculations. Figure 1 shows the Moran index plot of the green development performance for four years, with more than half of the cities within the 1st and 2nd quadrants showing high–high and low–low clustering characteristics. Figure 2 shows the urban market segmentation and the corresponding temporal and spatial quartiles of the green development performance for 2006 and 2019. It is clear from the figure that the degree of urban market segmentation in 2019 has increased compared to the past, and most cities in the central and northeast regions are located above the 0.5 range of market segmentation. At the same time, compared to 2006, the green development performance of these regions declined significantly in 2019, with most central and northeastern cities lying in a horizontal range below 0.24. Therefore, as the market fragmentation among cities increases, their green development performance decreases. It is especially important to accelerate market integration and eliminate market fragmentation to promote green development.

## 4. Empirical Results and Analysis

### 4.1. Baseline Model and Mediating Effects Regression Results

The results of the robust standard error regressions from the benchmark model and the mediating effects model, with the variables of the advanced level of industrial structure and the factor price distortion mechanism, are reported in Table 4, where CV stands for control variable and TP stands for turning point. Models (1) and (2) are the results of the double fixed effects regressions, including the primary and secondary terms of Mseg, respectively. The primary term coefficient of Mseg is significantly positive in model (1), as its primary term coefficient is significantly positive and its secondary term coefficient is significantly negative in model (2), while the model becomes insignificant when the tertiary term of market segmentation is introduced into model (3). This verifies hypothesis 1, in that there is an inverted U-shaped structure for the market segmentation and green development performance. The most important reason is that local enterprises are temporally able to improve the local green development performance due to the market segmentation originating from local protection, thus gaining a stronger cost advantage and monopoly profit source in the short run. Nevertheless, when the degree of market segmentation is increasing in the long run, there will be some obstacles to the free flow of resources and factors. Under these circumstances, resource factor allocation does not reach its optimal level of efficiency, while local protection allows for local enterprises to obtain profits in the market without participating in market competition, thus inhibiting enterprises from improving their long-term technology and product quality, thereby making them lose the ability to compete with neighboring regions in the market. 

Model (2) in Table 4 is the regression result of Equation (1) and the first step to testing the mediating effect, model (4) is the regression result of the mediating variable of the industrial structure advanced level of Equation (2), and model (5) is the introduction of the cubic term of the market segmentation variable Mseg to test the relationship between the advanced industrial structure and the market segmentation with the cubic term. In model (5), except for the significantly positive primary term of market segmentation, the secondary and tertiary terms are not significant. When it comes to model (4), it can be found that the primary and secondary terms of the market segmentation coefficients are significantly positive and negative, respectively. This result hints at the existence of an inverted U-shaped correlation between the market segmentation and local industrial structure upgrading. On the basis of these findings, it is argued that hypothesis 2 is verified. Model (6) is the result of the regression of Equation (3). The coefficient of the advanced level of industrial structure in model (6) is significantly positive. This result hints at the significant contribution of industrial structure upgrading to the local green development performance, while the primary and secondary terms of market segmentation can be found to be lower compared with those in model (2). This means that industrial structure upgrading accounts for a partial mediating effect. This indicates that market segmentation restricts the access of foreign enterprises and impedes the flow of factors, which makes local enterprises receive higher cost and profit advantages in the short term and significantly promotes the output and industrial structure upgrading of local enterprises. However, in the long term, as the degree of market segmentation and local protection increases, there are some obstacles to the competitiveness of the enterprises and the level of industrial structure, especially the lack of free flow and effective allocation of technology, talent, and resources, which, in turn, inhibits the green development performance. The regression result of the mediating variable factor price distortion is obtained in model (7), and the regression result after introducing the quadratic term of Mseg is obtained by following model (8). From the results of models (7) and (8), it is clear that no U-shaped correlation exists between the market segmentation and factor price distortions, but that market segmentation can significantly exacerbate the factor price distortions. This is mainly due to the local protection of market segmentation through administrative power to restrict the outflow of local factors and commodities, also while restricting the inflow of foreign commodities, factors, and firms, for the purpose of protecting the production of local firms. As a result, the free flow of the factors is prevented, thus making the factor prices constantly distorted, which is tested by hypothesis 3 in this study. Model (9) is the result of the regression in Equation (3). In model (9), the significantly negative factor price distortion coefficient hints at the significant inhibiting effect of the factor price distortion on the local green development performance, while the primary and secondary coefficients of the market segmentation are unchanged, though the secondary coefficient is lower compared to the coefficient in model (2), indicating that the factor price distortion accounts for a partial mediating effect. This also suggests that factor price distortions delay the inflection point of the market segmentation on the regional green development efficiency, suggesting that local governments can instead increase the degree of market segmentation by further increasing the local protection under a factor price distortion mechanism, allowing for local firms to continuously obtain lower factor costs, thus maintaining higher profits. The constant distortion of the factor prices will make the factor price market affect the profitability of each industry in a dysfunctional manner, and some high-energy-consumption and high-pollution industries still maintain a high profitability, preventing these enterprises from exiting the market and resulting in the loss of regional green development performance.

### 4.2. Regression Results of Dynamic Panel and Dynamic Spatial Panel Models

First, the dynamic panel data model eventually leads to the generation of a large bias from the traditional OLS estimation method, due to the presence of first-order lagged terms of Mseg, containing cete and thus correlating with the nuisance terms, as well as the existence of a two-way causality between the explanatory and explained variables, leading to endogeneity problems. Therefore, drawing on previous studies in the literature, this study uses a differential GMM and systematic GMM estimation for the dynamic panel model. At the same time, it takes into account the possible endogeneity of calculating the commodity market segmentation method with the explanatory variables. In this study, both the explanatory variables and the relevant control variables of the market segmentation are treated with a one-period lag [55,56,57]. Second, using a dynamic spatial panel lag model with time and city individual double fixed effects (due to the limitation of space, the results of the LM and Wald tests are not reported in this paper, and interested parties can contact the authors for them), the spatial spillover effects of the green development performance are estimated through conducting the LM, Wald, and Hausman tests. Due to the possibility of a bidirectional causal endogeneity between the explanatory and explained variables, considering dynamic SAR models, Elhorst (2012) estimated the dynamic spatial panel models using a differential GMM and found that such estimators may still be heavily biased. Kukenova and Monteiro (2009) extended Blundell and Bond’s (1998) systematic GMM for estimating dynamic spatial panel models and found that the systematic GMM estimators outperformed the differential GMM and reduced the parameter estimation error. With reference to existing research findings, the SGMM method is used in this study for the estimation of the robustness of the constructed dynamic spatial panel model, considering the model robustness based on the use of a quasi-great likelihood estimation [37,58,59].

As shown in Table 5, models (1) and (2) are the regression results of the general dynamic panel that were estimated using a differential GMM and systematic GMM, respectively, and the models both pass the serial correlation test of the nuisance terms, as well as the instrumental variable validity test. Accordingly, the time lag term of the green development performance regression is significantly positive in both ways, hinting at the generation of a significant inertia effect from green and low-carbon development in time with better path continuity, which means that accelerating the green and low-carbon process now has a strong pushing effect on future sustainable development. In terms of the market segmentation index, its significantly positive primary term coefficient and significantly negative secondary term coefficient imply the existence of an inverted U-shaped structure for the association between the green low-carbon development performance and market segmentation. The regression results of models (3) and (4) were estimated using quasi-great likelihood and systematic GMM methods for dynamic spatial panel models, respectively. It was found that the correlation coefficient of the spatial lagged term of the green development performance was significantly positive, indicating a positive spatial spillover effect in space. This may be due to the fact that local governments, while considering economic development, are also paying more attention to environmental benefits such as green and low-carbon effects, and there is an imitative learning effect for the green industrial policies or low-carbon emission reduction measures of neighboring or similar cities. The temporal lag term of green development performance (i.e., a better green development performance achieved by neighboring cities for the previous period) affects the region in the current period to a large extent. This is mainly due to the existence of market segmentation and local government competition, and city-to-city measures such as the learning of relevant industrial policies and the attraction of factor flows through imitation. When neighboring cities improve their green development level in the current period, they tend to attract limited resources and factors through administrative or financial incentives, and due to local protection and market segmentation, the factors and resources do not flow and allocate reasonably, thus inhibiting green development in the next period. When it comes to the explanatory variable Mseg, its significantly positive primary term coefficient and significantly negative secondary term coefficient are well-established evidence of the inverted U-shaped relationship between its market segmentation and green development performance, which still holds when based on the consideration of spatial correlation. Furthermore, by observing the inflection points, it can be found that the dynamic spatial panel model has earlier inflection points compared with the ordinary panel regressions that do not consider spatial correlation, which indicates that the dynamic spatial panel takes into account the spatial correlation between cities, so that it is highly possible that more comprehensive and realistic regression results are obtained with regards to the impact of market segmentation on the green low-carbon development performance.

### 4.3. Robustness Analysis

Table 6 shows the regression, with different spatial weight matrices, of the dynamic spatial panel model, to identify whether the dynamic spatial panel model constructed in this study is robust. From the results, it can be seen that both a quasi-great likelihood estimation and SGMM estimation, with varying spatial weight matrices, have the same sign as the estimation of the coefficients of the parameters under the spatial weight matrix of the shortest cross-city driving distance used in the main regression of this study, which means that the market segmentation and green development performance still maintain the inverted U-shaped relationship. Therefore, the dynamic spatial panel model constructed is also more robust.

### 4.4. Analysis of Direct and Indirect Effects

Changes in the degree of urban market segmentation, in the case of a spatial spillover effect, will not only affect the local green development performance but also affect the neighboring cities through a circular feedback effect and cause a number of adjustments. Therefore, market segmentation can be further classified as affecting the green development performance into direct and indirect effects [60]. The direct effect includes both the effect on the green development performance in the case of any change in the local city’s degree of market segmentation, as well as the spatial feedback effect, which is a circular process affecting neighboring cities with regards to the aspect of green and low-carbon development performance, which, in turn, affects the local city. The impact of changes in the local urban market segmentation on surrounding areas, with regards to the green development performance, is referred to as the indirect effect and also known as the spatial spillover effect. In consideration of the use of the dynamic spatial panel model herein, it is possible to subdivide the direct and indirect effects into long-term direct effects, long-term indirect effects, short-term direct effects, and short-term indirect effects in the time dimension.

The results of the direct and indirect effects, as shown in Table 7 and Table 8, are obtained through the decomposition of different spatial weight matrices under the great likelihood estimation of model (3) in Table 5, and models (1), (3), and (5) in Table 6. It can be found that short-term and long-term direct effects both show an inverted U-shaped structure, with the short-term effects weaker than the long-term effects. This is also consistent with the results of hypothesis 1. In the short run, indirect effects show an inverted U-shaped structure, but are found to have a positive U-shaped structure in the long run. This finding can be explained as follows: in the short term, when the local city increases the degree of market protection, the neighboring city also increases the degree of protection due to the imitative learning effect, which promotes the neighboring cities to have a better green development performance, but as the neighboring city continues to increase the degree of fragmentation, this spillover effect is offset by the efficiency loss caused by the fragmentation, thus inhibiting the green low-carbon development of the neighboring city. However, in the long run, the increasing degree of market segmentation in local cities prevents foreign enterprises and production factors from entering local cities, then only flowing to the neighboring cities where markets are highly accessible with a high degree of openness, so that the spatial spillover effect of the market segmentation shows a positive U-shaped structure, first decreasing, and then increasing in the long run. Of course, the level of this spillover effect is low because there is a relationship between the imitation and competition among cities, and the cities will adjust their corresponding competitive response in time. Most cities still have a competitive pattern of “one glory is prosperous; one loss is all”. In terms of both the aggregate indirect and long-term and short-term direct effects, there is an inverted U-shaped structure for the changes of the green development performance of cities, with an increase in the degree of market segmentation. To be specific, it increases and then decreases, with the short-term effects weaker than the long-term effects. Therefore, in the long run, accelerating the construction of a large unified market for the whole nation, reducing market segmentation, and eliminating local protection are key factors in the promotion of the long-term green and low-carbon sustainable development of cities.

### 4.5. Heterogeneity in This Analysis

China’s reform and opening up resulted in the rapid economic growth of urban areas. However, the subsequent regional development strategies that were carried out vary due to their different conditions and comparative advantages. For example, coastal regions—such as the Yangtze River and the Pearl River Deltas—have taken the lead in economic development. Likewise, there are various urban clusters—such as Hebei, Tianjin, and Beijing—that adopt a synergistic and integrated development strategy. Central China’s inland and western cities, however, do not have the same geographic advantages, and their development decreases the further away they are from eastern coastal areas. Therefore, further analysis is needed to determine how market segmentation affects the green development performance differently in eastern, central, and western cities. China is a vast country with a large population, and for each city, the influence of resource endowment on its development strategies, industrial layout, and market segmentation differs [61]. We take into account the heterogeneity of eastern, central, and western regions by considering whether a city is “resource-based” or “non-resource-based”, as classified in the document “National Sustainable Development Plan for Resource-based Cities” released by the State Council in 2013.

Models (1) and (5) in Table 9 and model (1) in Table 10 show the results of the regressions with static panel two-way fixed effects for the sample data, divided into eastern, central, and western cities, and with a robust standard error estimation. The regression results of the three regions under the static panel model show an inverted U-shaped structure for the influence of the market segmentation and green development performance in both eastern and central cities. This impact is not significant in western cities, which may be because the endogeneity was not completely eliminated. In addition to fixed effects such as city and time, the geographical location and climate, as well as any cultural differences arising from the gathering of ethnic minorities in the western region, make the populations, economies, and industries all appear larger than those in the eastern and central regions. At the same time, there are more central fiscal transfers in the western region, which will have a similar impact on the regional economic development and green emission reduction, and a static panel estimation does not eliminate these stochastic perturbations well. However, the GMM approach—by introducing instrumental variables—can solve the model endogeneity problem well. Table 9’s models (2), (3), (6), and (7), and Table 10’s models (2) and (3) are estimated using differential GMM and systematic GMM methods, respectively, for the sample data divided into eastern, central, and western cities. The random disturbance term autocorrelation and instrumental variables’ validity are tested, indicating the reasonability of the model settings and estimation methods, as well as of the selection of the instrumental variables. The model regression results also reveal that the green development performance in western, central, and eastern regions receives a significant inverted U-shaped effect from market segmentation, regardless of the results of the differential GMM or systematic GMM estimation. The regressions are more robust than the static panel model after the introduction of instrumental variables to address the endogeneity in western cities.

Table 9’s models (4) and (8) and Table 10’s model (4) show the regression results of the sample data from the western, central, and eastern regions using a dynamic spatial panel SGMM. The regression results show that market segmentation affects the green development performance in eastern, central, and western cities with an inverted U-shaped structure, and an inflection point at 0.5212, 0.4324, and 0.3258 for each region, respectively. This because central and western cities perform better than eastern cities in industrial and economic development. Even when the market segmentation reaches an inflection point that inhibits the advancement of industrial structure, the loss of emission reductions caused by the reduction of the industrial structure in eastern regions is lower than that of central and western cities. Moreover, it can be seen from the spatial correlation coefficient that central cities have stronger positive spatial effects than eastern cities, all significant at the 1% level, hinting at the necessity for central cities to accelerate their green and low-carbon development and give play to their urban–regional linkage effects, in order to promote an overall regional green development. It can be noticed in the examination of the western cities that their spatial correlation coefficient is negative at the 1% significance level, which may be associated with the simplified industrial structure and lower level of economic development in western cities, with more economic structures dominated by agriculture, industry, and resource extraction categories. Most of the local government’s fiscal revenue also comes from these industries, so even with the pressure of an ecological and environmental assessment, it is difficult for the government to release local protection, and to rectify and adjust high-energy-consumption and high-pollution enterprises. According to the “pollution paradise” hypothesis [62], although these highly polluting and energy-consuming enterprises cause great pressure to the environment, they can boost the economic development level and tax revenue of western cities, and the western city governments will be willing to bear this ecological and environmental pressure to accept the investment and relocation of these enterprises.

As shown in Table 11, the sample data has regressed from the perspective of re-source-based and non-resource-based cities. According to the regression results given in the table, the market segmentation in resource-based cities shows a significant inverted U-shaped structure in relationship with their green and low-carbon development, but non-resource-based cities are not found to receive a significant effect even after considering endogeneity. This may be due to the principle that market segmentation exerts its influence on regional green development through limiting the free flow of factors, increasing the distortion of the factor prices, and reducing the rate of the resource allocation, with a higher level of market segmentation undermining the upgrading of local industrial structure [63]. The “resource curse” theory states that resource-rich regions or countries often do not have sufficiently favorable conditions for economic growth, and this can become a constraint [64]. These resource-rich regions tend to increase their resource dependence, because natural resources can bring considerable “resource dividends” in the short term, causing governments and people to immerse themselves in the short-term benefits of natural resource exploitation activities and place a greater emphasis on the protection of traditional high-pollution and energy-consuming industries, such as resource extraction [65]. Market segmentation restricts the inflow of foreign resources, enterprises, and commodity factors through administrative means. This protects the monopoly production of high-energy-consumption and high-pollution firms and other local resource exploitation enterprises, and substantially reduces the productivity and profits of enterprises through channels such as R&D innovation, transformation, and upgrading. The effect that this brings is more inhibiting to the overall industrial upgrading of urban areas. Therefore, resource-based cities are motivated to prevent resource exploitation, and high-energy-consumption and high-polluting industries. Due to the lack of resource endowment advantages, non-resource-based cities, in the pursuit of urban development, have to optimize their industrial layout and provide an endogenous impetus for economic growth through industrial upgrading. Therefore, the impact of local protection on industrial upgrading in non-resource-based cities may not be too high. Industrial structure upgrading therefore plays a non-significant role in enabling market segmentation to the affect the green development performance.

## 5. Conclusions and Implications

This study provides a new research perspective for China to improve its environmental pollution and promote the construction of a large national unified market by examining the impact of market segmentation on green development performance, through the angle of cities. We further explore the mediating effects of the industrial structure and factor price distortions between the two. The main findings are as follows.

(1) Cities have a clear temporal and spatial path dependence on the green development performance. First, the time lag term of the green development performance is significantly positive at 1%. This reflects the improving sustainability of green low-carbon development and the necessity for China to adhere to a green low-carbon sustainable development strategy. Second, the spatial lag term of the green development performance is significantly positive at 1%, reflecting that it is the positive spatial spillover effects which create a “win some, lose some” duality. Therefore, China must accelerate its inter-regional cooperation and linkage with the formulation of environmental protection and emission reduction policies, strengthen its regional collaboration, and establish an integrated prevention and control mechanism across its administrative regions. Third, the time lag term of the green development performance is significantly negative at 1%, mainly due to local protection and serious regional market segmentation. This inhibits the green development performance of neighboring regions by restricting the outflow of factors and resources; therefore, there is a need to accelerate the market integration and eliminate the efficiency loss caused by the market segmentation.

(2) Our empirical findings suggest that the market segmentation stemming from local protection has a significant inverted U-shaped structure in relationship with the green development performance. Although a certain degree of market fragmentation can significantly improve the local green development performance, the “helping hand” brought about by such local protection is not sustainable, and exerts an obvious inhibiting effect on the neighboring regions with regards to the aspect of green development. Moreover, in the long run, as the degree of the market segmentation intensifies, it will inhibit the local green development, and the “helping hand” of local governments will become the “destroying hand”. This also explains why, given the efficiency losses caused by market segmentation, local governments in China still provide local protection and Chinese markets are still segmented. This is because, in the game between local governments, the local financial and tax incentives brought about by fiscal decentralization, the assessment of the ecological and environmental protection indicators, the performance assessment of officials’ promotions, and the implementation of market segmentation and local protection to support the development of local enterprises and increase the local tax revenue, become the preferred strategy. In addition, this study finds that industrial structure upgrading can significantly contribute to green development performance, while factor price distortion can significantly inhibit it. In the long run, market segmentation inhibits the green development performance by inhibiting the industrial structural upgrading and exacerbating the factor price distortions, so that accelerating the market integration among Chinese regions not only promotes regional industrial structural upgrading, but also optimizes resource allocation and promotes an overall regional efficiency.

(3) The heterogeneity analysis found that from the eastern, central, and western cities, although the market segmentation and green development performance are always found to have an inverted U-shaped correlation, the market segmentation value corresponding to the inflection point ranks 1st in the eastern cities, 2nd in the central cities, and 3rd in the western cities. This is due to the differences among the three regions in their levels of advanced industrial structure. Second, from the spatial correlation coefficients, both the central and eastern cities have positive spatial spillover effects, which are stronger in the central cities. However, the spatial correlation coefficients are significantly negative in the western cities, mainly because these western cities have a single industrial structure and insufficient factor endowment advantages. When the neighboring cities rectify the high-energy-consuming and high-polluting industries, they accept the relocated industries from the neighboring cities due to local development and tax revenue, which inhibits the local green development performance. However, due to the theoretical hypothesis of the “resource curse”, market segmentation still affects resource-based cities through the aspect of their green development performance. This is from the perspective of resource-based and non-resource-based cities, (although the former has better resource endowment advantages). The effect is in a significant inverted U-shaped structure, but the impact on the non-resource-based cities is not significant. For both types of cities divided by the resource endowment, the significantly positive spatial spillover effect of the green development performance still shows the spatial correlation pattern of “Benefit to one means benefit to all, whereas harm to one means harm to all”.

Synthesizing the analysis of this study’s research, the following policy recommendations are proposed to promote green development in Chinese cities. (1) To promote the achievement of carbon peaking and carbon neutrality in China, it is necessary to develop the country as a whole by promoting environmental protection, energy saving, and emission reduction in each city, facilitating the integrated growth of the regional ecology. (2) In line with the phrase “United we stand, divided we fall,” it is important to accelerate the market integration and build a large unified market in China, eliminating market segmentation, promoting the optimal allocation of factors, and ensuring the free flow of resources. (3) Local governments should cooperate with one another to bring into play their comparative advantages, optimize the industrial layout, and accelerate the industrial structure transformation. (4) Due to the large regional development gap in China, its western cities have a lower level of industrial sophistication, and are less attractive to capital, labor, and other factors than central and eastern cities. Thus, it is difficult to accelerate the regional industrial structure upgrading due to the lack of factor resources, causing the phenomenon of “pollution rather than development” that may exist in western cities. Therefore, western cities not only need to accelerate their market integration and break the local protection, but also need China’s national financial assistance to increase its transfer payments and financial subsidies to the less developed western regions, in order to help the western cities transform and upgrade their industries, accelerating green and low-carbon development. (5) China has a vast area and rich resources. Due to the over-exploitation practices in the past, resource depletion and other difficulties are now issues undermining the further development of resource-based cities. To ensure their growth, it is more important to implement policies that support the resource-based and resource-depleted cities in the pursuit of optimization and transformation of their industrial structure, to increase the openness of cities, and to attract the inflow of surrounding factors and resources to promote urban transformation and development. (6) In September 2020, China proposed the dual carbon policy to peak carbon dioxide emissions before 2030 and achieve carbon neutrality by 2060. In April 2022, the “Opinions of the State Council of the Central Committee of the Communist Party of China on Accelerating the Construction of a Unified National Market” was promulgated. This signified that the elimination of market fragmentation and the construction of a green emission reduction system have become the focus of national attention, and local governments should promote city cooperation and improve the openness of their local markets.

## Figures and Tables

**Table 1:** Input–output tables.

Input Variables	Desired Output Variables	Non-Desired Output Variables
Capital (10,000 yuan)	GDP (10,000 yuan)	CO_2_ emissions (10,000 tons)
Labor force (10,000 people)		Industrial wastewater (10,000 tons)
Energy consumption (10,000 tons)		Industrial SO_2_ (10,000 tons)
		Industrial fume and dust (10,000 tons)

**Table 2:** Descriptive statistics.

Variables	Obs	Mean	St.D	Min	Max
lnaw	3948	10.3206	0.4068	8.1849	11.6524
ps	3948	0.3569	0.2382	0.0435	1
fd	3948	0.3965	0.0994	0.0853	0.8918
er	3948	0.0033	0.0014	0.0002	0.0124
oul	3948	0.0141	0.0178	0.00001	0.2070
Cete	3948	0.3923	0.2411	0.0479	1.2447
Mseg	3948	0.3660	0.1049	0.0409	0.8260
lnsqt	3948	7.0714	1.8090	1.6094	12.3880
ts	3948	0.9143	0.5015	0.0943	5.1683
dist	3948	0.5881	0.1625	0.0025	0.9920
gov	3948	0.1905	0.1368	0.0353	2.2794
lnpop	3948	5.8724	0.6952	2.8685	8.1362
infs	3948	15.6799	6.9103	1.3700	60.0700
lnprgdp	3948	10.0891	0.7776	7.8779	12.6433
edu	3948	4.5235	1.1413	0.7322	7.2120

**Table 3:** Global Moran’s I of market segmentation, carbon performance, and model selection.

Year	CeteI	Year	CeteI
2006	0.018 *** (4.588)	2013	0.030 *** (7.363)
2007	0.044 *** (10.340)	2014	0.025 *** (6.176)
2008	0.047 *** (10.847)	2015	0.025 *** (6.256)
2009	0.049 *** (11.380)	2016	0.023 *** (5.734)
2010	0.049 *** (11.381)	2017	0.032 *** (7.674)
2011	0.042 *** (9.795)	2018	0.034 *** (8.157)
2012	0.034 *** (8.068)	2019	0.035 *** (8.385)
**Model**	**AIC**	**BIC**	**Log-likelihood**
SAR	−4987.62	−4811.75	2521.81
SEM	−4950.32	−4856.10	2490.16
SDM	−4950.15	−4855.15	2490.08
Hausman test	chi2(14)	Prob ≥ chi2	
	62.12	0.0000	

Notes: Z values in brackets, “*”, “**”, and “***” indicate passing the significance test of * *p* < 0.1, ** *p* < 0.05, and *** *p* < 0.01.

**Table 4:** Regression results of the baseline and mediated effects models.

				Intermediary Variable M = ts			Intermediary Variable M = dist		
	(1)	(2)	(3)	(4)	(5)	(6)	(7)	(8)	(9)
	OLS	OLS	OLS	OLS	OLS	OLS	OLS	OLS	OLS
	Cete	Cete	Cete	ts	ts	Cete	dist	dist	Cete
Mseg	0.175 **	0.979 ***	0.791	2.150 ***	2.773 **	0.908 ***	0.0709 *	0.00306	0.979 ***
	[0.0654]	[0.2210]	[0.5998]	[0.3671]	[0.9423]	[0.1944]	[0.0309]	[0.1143]	[0.1927]
Msegsq		−0.886 ***	−0.499	−1.438 ***	−3.003	−0.838 ***		0.0769	−0.880 ***
		[0.2373]	[1.4466]	[0.3716]	[2.1896]	[0.2092]		[0.1216]	[0.2085]
Msegtq			−0.280		1.214				
			[1.1284]		[1.6527]				
ts						0.0326 **			
						[0.0120]			
dist									−0.0769 *
									[0.0364]
_cons	0.203	0.949	0.0198	19.35 ***	19.25 ***	0.296	−6.274 ***	−6.253 ***	0.468
	[1.0873]	[1.0640]	[1.0832]	[2.0660]	[2.0685]	[1.0115]	[0.5625]	[0.5623]	[1.0086]
City FE	Yes	Yes	Yes	Yes	Yes	Yes	Yes	Yes	Yes
Time FE	Yes	Yes	Yes	Yes	Yes	Yes	Yes	Yes	Yes
CV	Yes	Yes	Yes	Yes	Yes	Yes	Yes	Yes	Yes
TP		0.5525		0.7476		0.5418			0.5563
*N*	3948	3948	3948	3948	3948	3948	3948	3948	3948
adj. *R*^2^	0.6891	0.6890	0.6904	0.8644	0.8644	0.6895	0.8582	0.8582	0.6893
*AIC*	−4934.0	−4933.0	−4948.6	−2425.5	−2424.3	−4939.0	−11,145.6	−11,144.3	−4935.9
*BIC*	−4852.3	−4851.4	−4854.4	−2337.5	−2330.1	−4851.0	−11,070.2	−11,062.7	−4848.0

Notes: robust standard errors in brackets, “*”, “**”, and “***” indicate passing the significance test of * *p* < 0.05, ** *p* < 0.01, and *** *p* < 0.001.

**Table 5:** Regression results of dynamic panel and dynamic spatial panel models.

	(1)	(2)	(3)	(4)
	Diff-GMM	SYS-GMM	Dynamic SAR-MLE(W1)	Dynamic SAR-SGMM(W1)
	Cete	Cete	Cete	Cete
L.Cete	0.554 ***	0.570 ***	0.6128 ***	0.559 ***
	[0.0027]	[0.0020]	[0.0144]	[0.0020]
W×Cete			0.4000 ***	2.119 ***
			[0.1191]	[0.0383]
W×L.Cete			−0.5097 **	−0.641 ***
			[0.2084]	[0.0293]
Mseg	0.794 ***	0.396 ***	0.5008 ***	0.241 ***
	[0.0431]	[0.0296]	[0.1684]	[0.0341]
Msegsq	−0.657 ***	−0.261 ***	−0.5392 ***	−0.331 ***
	[0.0486]	[0.0331]	[0.1805]	[0.0398]
_cons	−1.524 ***	−0.72 ***		−1.079 ***
	[0.216]	[0.017]		[0.0209]
TP	0.6043	0.7586	0.4644	0.364
City FE	Yes	Yes	Yes	Yes
Time FE	Yes	Yes	Yes	Yes
CV	Yes	Yes	Yes	Yes
	−7.7365	−7.7031		−7.6987
AR(1) *[P]*	[0.0000]	[0.0000]		[0.0000]
	1.614	1.6396		1.5689
AR(2) *[P]*	[0.1065]	[0.1011]		[0.1167]
	270.0106	268.4702		269.7069
*Sargan[P]*	[0.9655]	[1.0000]		[1.0000]
*N*	3666	3666	3666	3666

Notes: standard errors in brackets, “*”, “**”, and “***” indicate passing the significance test of * *p* < 0.1, ** *p* < 0.05, and *** *p* < 0.01.

**Table 6:** Regression results of dynamic spatial panel models.

	(1)	(2)	(3)	(4)	(5)	(6)
	Dynamic SAR-MLE (W2)	Dynamic SAR-SGMM(W2)	Dynamic SAR-MLE (W3)	Dynamic SAR-SGMM(W3)	Dynamic SAR-MLE (W4)	Dynamic SAR-SGMM(W4)
	Cete	Cete	Cete	Cete	Cete	Cete
L.Cete	0.6126 ***	0.561 ***	0.6133 ***	0.569 ***	0.6131 ***	0.551 ***
	[0.0144]	[0.0018]	[0.0143]	[0.0020]	[0.0144]	[0.0019]
W×Cete	0.3312 ***	1.208 ***	0.0310 *	0.115 ***	0.4020 ***	2.010 ***
	[0.1064]	[0.0229]	[0.0179]	[0.0037]	[0.1130]	[0.0286]
W×L.Cete	−0.5459 ***	−0.509 ***	−0.0427 **	−0.0205 ***	−0.5109 ***	−0.592 ***
	[0.1639]	[0.0225]	[0.0210]	[0.0025]	[0.1899]	[0.0324]
Mseg	0.5083 ***	0.324 ***	0.3851 **	0.420 ***	0.5017 ***	0.256 ***
	[0.1683]	[0.0440]	[0.1890]	[0.0364]	[0.1683]	[0.0377]
Msegsq	−0.5500 ***	−0.369 ***	−0.4255 **	−0.484 ***	−0.5406 ***	−0.364 ***
	[0.1805]	[0.0488]	[0.2020]	[0.0426]	[0.1805]	[0.0418]
_cons		−0.788 ***		−0.649 ***		−0.983 ***
		[0.0249]		[0.0252]		[0.0263]
TP	0.4621	0.439	0.4525	0.4339	0.464	0.3516
City FE	Yes	Yes	Yes	Yes	Yes	Yes
Time FE	Yes	Yes	Yes	Yes	Yes	Yes
CV	Yes	Yes	Yes	Yes	Yes	Yes
		−7.6826		−7.7192		−7.7035
AR(1) *[P]*		[0.0000]		[0.0000]		[0.0000]
		1.6417		1.6224		1.5025
AR(2) *[P]*		[0.1007]		[0.1047]		[0.1330]
		268.3943		265.4121		257.7253
*Sargan[P]*		[1.0000]		[1.0000]		[1.0000]
*N*	3666	3666	3666	3666	3666	3666

Notes: standard errors in brackets, “*”, “**”, and “***” indicate passing the significance test of * *p* < 0.1, ** *p* < 0.05, and *** *p* < 0.01.

**Table 7:** Estimation of direct and indirect effects under different spatial weight matrices.

	W1				W2			
	(1)	(2)	(3)	(4)	(5)	(6)	(7)	(8)
	Short-Term Direct Effects	Short-Term Indirect Effects	Long-Term Direct Effects	Long-Term Indirect Effects	Short-Term Direct Effects	Short-Term Indirect Effects	Long-Term Direct Effects	Long-Term Indirect Effects
	Cete	Cete	Cete	Cete	Cete	Cete	Cete	Cete
Mseg	0.5188 ***	0.3759	1.3386 ***	−0.2361	0.5260 ***	0.2788 *	1.3588 ***	−0.4594 **
	[0.1616]	[0.2286]	[0.4168]	[0.3270]	[0.1614]	[0.1638]	[0.4168]	[0.2199]
Msegsq	−0.5581 ***	−0.4042 *	−1.4399 ***	0.2541	−0.5687 ***	−0.3011 *	−1.4693 ***	0.4972 **
	[0.1689]	[0.2440]	[0.4357]	[0.3470]	[0.1689]	[0.1751]	[0.4360]	[0.2328]
*N*	3666				3666			

Notes: standard errors in brackets, “*”, “**”, and “***” indicate passing the significance test of * *p* < 0.1, ** *p* < 0.05, and *** *p* < 0.01.

**Table 8:** Estimation of direct and indirect effects under different spatial weight matrices.

	W3				W4			
	(1)	(2)	(3)	(4)	(5)	(6)	(7)	(8)
	Short-Term Direct Effects	Short-Term Indirect Effects	Long-Term Direct Effects	Long-Term Indirect Effects	Short-Term Direct Effects	Short-Term Indirect Effects	Long-Term Direct Effects	Long-Term Indirect Effects
	Cete	Cete	Cete	Cete	Cete	Cete	Cete	Cete
Mseg	0.4044 **	0.4044 **	1.0462 **	−0.0295	0.5198 ***	0.3759 *	1.3420 ***	−0.2425
	[0.1810]	[0.1810]	[0.4682]	[0.0505]	[0.1615]	[0.2187]	[0.4170]	[0.3032]
Msegsq	−0.4458 **	−0.4458 **	−1.1533 **	0.0328	−0.5596 ***	−0.4047 *	−1.4448 ***	0.2612
	[0.1887]	[0.1887]	[0.4881]	[0.0543]	[0.1689]	[0.2336]	[0.4360]	[0.3220]
*N*	3666				3666			

Notes: standard errors in brackets, “*”, “**”, and “***” indicate passing the significance test of * *p* < 0.1, ** *p* < 0.05, and *** *p* < 0.01.

**Table 9:** Heterogeneity analysis regression results.

	Eastern Cities				Central Cities			
	(1)	(2)	(3)	(4)	(5)	(6)	(7)	(8)
	OLS	Diff-GMM	SYS- GMM	Dynamic SAR-SGMM(W1)	OLS	Diff- GMM	SYS- GMM	Dynamic SAR-SGMM(W1)
	Cete	Cete	Cete	Cete	Cete	Cete	Cete	Cete
L.Cete		0.347 ***	0.516 ***	0.531 ***		0.447 ***	0.529 ***	0.503 ***
		[0.0186]	[0.0210]	[0.0147]		[0.0126]	[0.0185]	[0.0140]
W×Cete				1.750 ***				2.049 ***
				[0.2794]				[0.1692]
W×L.Cete				−0.521 ***				−1.114 ***
				[0.1965]				[0.1618]
Mseg	0.863 *	0.937 ***	1.085 ***	1.202 ***	2.015 ***	0.973 ***	1.013 ***	0.723 ***
	[0.4801]	[0.3479]	[0.3389]	[0.3764]	[0.3503]	[0.2394]	[0.2328]	[0.2100]
Msegsq	−0.804 *	−0.996 ***	−1.001 ***	−1.153 ***	−2.108 ***	−1.135 ***	−1.033 ***	−0.836 ***
	[0.4552]	[0.3524]	[0.3320]	[0.3859]	[0.3836]	[0.2870]	[0.3124]	[0.2592]
_cons	4.544 *	9.078 ***	0.122	−1.316 ***	−2.207	2.374 **	−0.419 *	−1.173 ***
	[2.7090]	[1.2204]	[0.3017]	[0.3270]	[1.9042]	[1.0264]	[0.2445]	[0.2304]
TP	0.5367	0.4704	0.542	0.5212	0.4993	0.4286	0.4903	0.4324
City FE	Yes	Yes	Yes	Yes	Yes	Yes	Yes	Yes
Time FE	Yes	Yes	Yes	Yes	Yes	Yes	Yes	Yes
CV	Yes	Yes	Yes	Yes	Yes	Yes	Yes	Yes
		−4.7161	−4.8668	−4.8844		−4.1641	−4.3052	−4.1487
AR(1) *[P]*		[0.0000]	[0.0000]	[0.0000]		[0.0000]	[0.0000]	[0.0000]
		1.1674	1.6378	1.616		0.52609	0.66333	0.93317
AR(2) *[P]*		[0.2431]	[0.1015]	[0.1061]		[0.5988]	[0.5071]	[0.3507]
		84.32526	75.16387	67.1353		84.18583	86.54511	83.50581
*Sargan[P]*		[1.0000]	[1.0000]	[1.0000]		[1.0000]	[1.0000]	[1.0000]
adj. *R*^2^	0.7267				0.6852			
*N*	1400	1200	1300	1300	1386	1188	1287	1287

Notes: robust standard errors in brackets, “*”, “**”, and “***” indicate passing the significance test of * *p* < 0.05, ** *p* < 0.01, and *** *p* < 0.001.

**Table 10:** Heterogeneity analysis regression results.

	Western Cities			
	(1)	(2)	(3)	(4)
	OLS	Diff-GMM	SYS-GMM	Dynamic SAR-SGMM(W1)
	Cete	Cete	Cete	Cete
L.Cete		0.499 ***	0.543 ***	0.554 ***
		[0.0253]	[0.0212]	[0.0255]
W×Cete				−1.405 ***
				[0.3215]
W×L.Cete				1.226 ***
				[0.4468]
Mseg	0.281	1.180 ***	1.340 ***	0.703 **
	[0.5128]	[0.4326]	[0.3516]	[0.3250]
Msegsq	−0.208	−1.363 **	−1.960 ***	−1.079 **
	[0.7561]	[0.5975]	[0.5481]	[0.5178]
_cons	−1.629	−4.660 ***	−0.582 **	−0.367
	[2.6008]	[1.6759]	[0.2570]	[0.3969]
TP	0.6755	0.4329	0.3418	0.3258
City FE	Yes	Yes	Yes	Yes
Time FE	Yes	Yes	Yes	Yes
CV	Yes	Yes	Yes	Yes
		−4.1312	−3.9273	−4.1394
AR(1) *[P]*		[0.0000]	[0.0001]	[0.0000]
		−0.22544	−0.20016	−0.23941
AR(2) *[P]*		[0.8216]	[0.8414]	[0.8108]
		68.93893	55.67243	56.32576
*Sargan[P]*		[1.0000]	[1.0000]	[1.0000]
adj. *R*^2^	0.5680			
*N*	1162	996	1079	1079

Notes: standard errors in brackets, “*”, “**”, and “***” indicate passing the significance test of * *p* < 0.1, ** *p* < 0.05, and *** *p* < 0.01.

**Table 11:** Heterogeneity analysis regression results.

	Resource-Based Cities				Non-Resource-Based Cities			
	OLS	Diff-GMM	SYS- GMM	Dynamic SAR-SGMM(W1)	OLS	Diff-GMM	SYS- GMM	Dynamic SAR-SGMM(W1)
	Cete	Cete	Cete	Cete	Cete	Cete	Cete	Cete
L.Cete		0.592 ***	0.611 ***	0.621 ***		0.483 ***	0.563 ***	0.533 ***
		[0.0125]	[0.0146]	[0.0111]		[0.0053]	[0.0067]	[0.0081]
W×Cete				1.769 ***				2.199 ***
				[0.1033]				[0.1296]
W×L.Cete				−1.159 ***				−0.435 ***
				[0.1019]				[0.0930]
Mseg	0.748 ***	0.814 ***	0.605 ***	0.403 ***	1.021 ***	−0.0445	0.210 *	0.122
	[0.2706]	[0.1633]	[0.0757]	[0.0854]	[0.3645]	[0.1354]	[0.1082]	[0.1258]
Msegsq	−0.494 *	−0.701 ***	−0.529 ***	−0.445 ***	−1.117 ***	0.261	−0.0855	−0.183
	[0.2904]	[0.1842]	[0.1039]	[0.1010]	[0.3868]	[0.1615]	[0.1222]	[0.1595]
_cons	−0.211	0.626	−0.692 ***	−0.837 ***	−0.0724	−6.456 ***	−0.139 **	−0.471 ***
	[1.3461]	[0.7398]	[0.0837]	[0.0898]	[1.7195]	[0.6441]	[0.0611]	[0.0726]
TP	0.7571	0.5806	0.5718	0.4528	0.457			
City FE	Yes	Yes	Yes	Yes	Yes	Yes	Yes	Yes
Time FE	Yes	Yes	Yes	Yes	Yes	Yes	Yes	Yes
CV	Yes	Yes	Yes	Yes	Yes	Yes	Yes	Yes
		−4.194	−4.1917	−4.1156		−6.46	−6.5023	−6.5748
AR(1) *[P]*		[0.0000]	[0.0001]	[0.0000]		[0.0000]	[0.0000]	[0.0000]
		0.42692	0.49729	0.44124		1.4391	1.6235	1.576
AR(2) *[P]*		[0.6694]	[0.6190]	[0.6590]		[0.1501]	[0.1045]	[0.1150]
		87.44789	89.6426	87.28543		154.1821	147.4394	145.8696
*Sargan[P]*		[1.0000]	[1.0000]	[1.0000]		[1.0000]	[1.0000]	[1.0000]
adj. *R*^2^	0.5602				0.7105			
*N*	1596	1368	1482	1482	2352	2016	2184	2184

Notes: robust standard errors in brackets, “*”, “**”, and “***” indicate passing the significance test of * *p* < 0.05, ** *p* < 0.01, and *** *p* < 0.001.

## Data Availability

Data sharing is not applicable to this article.
